# Baseline impulsivity may moderate L-DOPA effects on value-based decision-making

**DOI:** 10.1038/s41598-019-42124-x

**Published:** 2019-04-04

**Authors:** Johannes Petzold, Annika Kienast, Ying Lee, Shakoor Pooseh, Edythe D. London, Thomas Goschke, Michael N. Smolka

**Affiliations:** 1 Department of Psychiatry and Neuroimaging Center, Technische Universität Dresden, Dresden, Germany; 2 Department of Experimental Psychology, University of Oxford, Oxford, United Kingdom; 3 Freiburg Center for Data Analysis and Modeling, Albert-Ludwigs-Universität Freiburg, Freiburg, Germany; 4 Department of Psychiatry and Biobehavioral Sciences, Department of Molecular and Medical Pharmacology and the Brain Research Institute, University of California at Los Angeles, Los Angeles, CA USA; 5 Department of Psychology and Neuroimaging Center, Technische Universität Dresden, Dresden, Germany

## Abstract

Research has indicated a major role of dopamine in decision-making processes, but the underlying mechanisms remain largely unknown due to inconsistency in effects of dopaminergic drugs. To clarify the impact of dopamine on impulsive choice, we administered 150 mg L-DOPA to 87 healthy adults in a randomized, placebo-controlled, double-blind, crossover study, evaluating performance in four value-based decision-making tasks. We predicted that baseline impulsivity would moderate L-DOPA effects. In support of our hypothesis, L-DOPA had no main effect on impulsive choice, but reduced risk-seeking for gains in more-impulsive subjects. Because L-DOPA effects may be influenced by body weight, we repeated our analyses on data from half of the sample (n = 44) with lower weight, anticipating a stronger effect. In addition to the effect on risk-seeking for gains, low-weight participants also exhibited baseline-dependent effects of L-DOPA on loss aversion and delay discounting. Our results are consistent with the hypothesis of an inverted U-shaped dopamine function in which both low and high extremes of dopamine signaling are associated with high-impulsive choice. Consideration of differential baseline impulsivity and body weight may resolve previous seemingly paradoxical pharmacological results and might deepen our understanding of dopaminergic mechanisms underlying impulsivity.

## Introduction

Value-based decision-making is a complex cognitive process that requires balancing potential rewards against their potential costs, and incorporates the probability of obtaining them and/or the delay until gratification. Preference for immediately available small rewards over larger but delayed ones (delay discounting) and for probabilistic rewards over smaller but certain ones (risk-seeking), and insensitivity to loss reflect different domains of impulsive decision-making^1^, which characterize psychiatric disorders, such as attention-deficit hyperactivity disorder (ADHD)^2^, gambling disorder^3,4^, substance use disorders^3^ and bipolar disorders^5,6^. A central, highly complex role of dopamine has been implicated in these decision-making processes^7,8^. Drugs that augment dopaminergic function offer therapeutic benefit for ADHD (e.g., methylphenidate, amphetamine) and tobacco use disorder (e.g., bupropion). Yet administration of dopamine agonists can lead to impulsive behavior, such as pathological gambling and hypersexuality^9^.

One approach to delineate the role of dopamine in impulsive choice is through pharmacological studies in healthy humans^7^. Boosting dopaminergic signaling via d-amphetamine (20 mg) decreased delay discounting^10^, and administration of the dopamine D2 receptor antagonist quetiapine (150 mg) promoted risk-seeking (in males but not females) in a gambling task^11^. These findings may partly explain why dopamine-enhancing drugs offer therapeutic benefit for patients with ADHD. Indeed, methylphenidate reduced delay discounting in children with ADHD who received actual money in real time^12^.

However, as 150 mg quetiapine is probably insufficient to occupy dopamine D2 receptors^13^ and as quetiapine has multiple actions^14^, risk-seeking may have been promoted by effects on non-dopaminergic neurotransmission. Other contradictory findings include lack of effects of methylphenidate on hypothetical delay discounting and risk-seeking as assessed with a probability discounting for gains task in children with ADHD^12^, and of d-amphetamine (20 mg) on risk-seeking for gains in healthy adults^10,15^. Moreover, a decrease in delay discounting by 20 mg d-amphetamine was not replicated^15^; and 10 mg d-amphetamine^10^ as well as other dopamine-enhancing drugs, such as the dopamine D2/D3 receptor agonist pramipexole (0.25 or 0.5 mg)^16^ or the dopamine reuptake inhibitor bupropion (150 or 300 mg)^15^, did not affect either delay discounting or risk-seeking for gains. These results are comparable to other findings: haloperidol (1.5 mg) did not affect delay discounting^17^, and L-DOPA had no influence on risk-seeking for gains (100 mg)^18^, risk-seeking for losses or loss aversion as assessed with a mixed gambles task (150 mg)^19^. In other studies, L-DOPA (150 mg) even increased delay discounting^17^ and risk-seeking for gains (only in low-weight subjects in Rigoli *et al*.)^19,20^. Consistent with these findings, the dopamine D2 receptor antagonist metoclopramide (10 mg)^21^ and the dopamine D2/D3 receptor antagonist amisulpride (400 mg)^22^ reduced delay discounting.

In summary, drugs that increase dopamine signaling as well as those that reduce it reportedly both boosted and diminished impulsive choice, in line with no net effect of dopamine on value-based decision-making. Use of different tasks and drugs that affect different components of dopaminergic signaling (e.g., dopamine release, reuptake, receptor binding) may have contributed to inconsistency. A plausible explanation for the paradoxical effects of dugs affecting dopamine signaling could also be an inverted U-shaped function, where low and high extremes of dopamine signaling are linked to worse cognition^23,24^, including decision-making performance^25^. Applied to value-based decision-making, individuals with suboptimal dopamine signaling would become less impulsive with dopamine-enhancing drugs, whereas those with optimal dopaminergic signaling would get overdosed and make more impulsive choices. This working model is supported by a study using scores on the Barratt Impulsiveness Scale (BIS-11) as a metric for baseline impulsivity^26^. Administration of tolcapone, an inhibitor of the dopamine-degrading enzyme catechol-O-methyltransferase (COMT), decreased delay discounting in high-impulsive participants, whereas low-impulsive participants exhibited smaller declines or enhancements in delay discounting^26^. An association between trait impulsivity and dopamine signaling has been observed using positron emission tomography (PET). Higher BIS-11 impulsivity and lower striatal D2/D3 receptor availability were found in recently abstinent methamphetamine-dependent subjects compared to healthy controls with BIS-11 related to D2/D3 receptor availability across both groups^27^. Along this line, higher BIS-11 scores also were associated with higher striatal dopamine transporter availability (consistent with lower dopaminergic tone) in healthy men^28^. These findings may reflect the ascending limb of an inverted U-shaped function describing the relationship between dopamine activity (increasing from left to right on x-axis) and BIS-11 impulsivity (decreasing from bottom to top on y-axis), assuming that the descending limb did not emerge since acutely overdosed individuals were not tested. Of note, contrasting data have also been reported, although limited to BIS subscales in small-sample studies^29,30^.

To investigate whether baseline impulsivity moderates L-DOPA effects on value-based decision-making, we tested 87 healthy adults on four facets of impulsive choice in a randomized, placebo-controlled, double-blind, crossover study. We predicted that participants with stronger impulsivity on the BIS-15 would become less impulsive after L-DOPA administration, whereas those with lower impulsivity at baseline would then make more impulsive choices after receiving L-DOPA.

## Material and Methods

### Participants

This dataset was part of the project “Dopaminergic Modulation of Meta-Control Parameters and the Stability-Flexibility Balance” within the Collaborative Research Center 940 “Volition and Cognitive Control: Mechanisms, Modulators and Dysfunctions” (www.sfb940.de). The project combined a pharmacological challenge of the dopamine system with self-report measures, behavioral paradigms and neuroimaging. The Residents’ Registration office of Dresden provided a population sample (N = 15778) stratified by age (20–40 years) and sex. We screened interested residents (N = 1383) for eligibility using the following exclusion criteria: current or past neurological or mental disorders except for nicotine dependence (assessed with the Structured Clinical Interview for DSM-IV)^31^, recent use of alcohol (breath-alcohol analysis on intervention visits; Alcotest 6510, Dräger, Lübeck, Germany) or illicit drugs (urine test on first intervention visit; Kombi/DOA10-Schnelltest, MAHSAN Diagnostika, Reinbek, Germany), visual impairment (visual acuity < 0.8 with correction) and contraindications to L-DOPA or magnetic resonance imaging. Eligible individuals were invited to a baseline visit when they became acquainted with a computerized value-based decision-making battery and completed the BIS-15, which measures trait impulsivity (range of scores: 15 to 60, higher scores indicating stronger impulsivity)^32^. Six hundred eleven participants completed the baseline visit, of which 103 attended the intervention visits, which were at least 7 days apart (mean = 12.6, SD = 8.7). Other individuals in the sample participated in another project: “Serotonergic Modulation of Meta-Control Parameters^33^.” Eighty-seven participants completed the value-based decision-making battery after both interventions (for participant characteristics see Table 1), whereas 16 participants dropped out due to technical issues (n = 2), erroneous drug manipulation (n = 3), schedule difficulties (n = 3) and adverse events (panic attack symptoms [n = 4], vomiting [n = 1], unspecific discomfort [n = 3]).

**Table 1:** Participant characteristics.

	All subjects	Low-weight subjects
Number of participants	87	44
Gender (males, females)	65, 22	25, 19
Age [years] at baseline (mean ± SD, median, range)	35.91 ± 3.80, 35.92, 29–42	35.45 ± 3.80, 35.61, 30–42
Weight [kg] at L-DOPA visit (mean ± SD, median, range)	80.19 ± 14.18, 80.50, 49–128	69.84 ± 8.65, 72.35, 49–81
BIS-15 total score (mean ± SD, median, range)	29.69 ± 4.95, 30.00, 17–44	29.05 ± 4.91, 28.50, 21–40

### Drug intervention

The dopamine precursor L-DOPA was administered in a randomized, placebo-controlled, double-blind, crossover study. To equalize the absorption of L-DOPA, which is influenced by food^34^, participants arrived in the morning after having fasted overnight. Upon arrival, they received butter biscuits (~120 kcal) and dextrose tablets (~17 kcal per hour) to reduce side effects of L-DOPA. They took 187.5 mg Madopar in tablet form (150 mg L-DOPA + 37.5 mg benserazide, a peripherally-acting DOPA decarboxylase inhibitor; Roche, Grenzach-Wyhlen, Germany) or a matched placebo and completed tasks that are not part of the present work. A booster dose of 93.75 mg Madopar in tablet form (75 mg L-DOPA + 18.75 mg benserazide) or a matched placebo was administered 100 min after the first dose and 50 min prior to the value-based decision-making battery. The study design enabled a maximum L-DOPA level at the time of task execution^35^ and comparison to results of previous studies in which L-DOPA was administered in doses between 100^18^ and 150 mg^17,19,20^.

### Value-based decision-making test battery

The test battery^36^ included four tasks, measuring different facets of impulsive choice: Delay Discounting, Probability Discounting for Gains, Probability Discounting for Losses and Mixed Gambles. Differences between the published battery^36^ and versions of the tasks used here were previously described^37^. On each task, participants chose between two simultaneously presented offers, which randomly appeared on the left or right side of a computer screen. There was no time limit for making decisions, and the chosen offer was highlighted with a frame. Participants were not informed about outcomes, but were instructed that after each task, one trial would be randomly selected and paid immediately or with the actual delay/probability according to the given choice.

The Delay Discounting task consisted of 30 trials in which €5–30 were offered with delays of 3 days, 1 week, 2 weeks, 1 month, 2 months, 6 months or 1 year. Either a smaller, immediately available amount or a larger amount, available after one of the above-mentioned delays, could be chosen (e.g., getting €7 now or €10 in 1 week). The task measures the extent to which individuals discount rewards as a function of delay; a higher k indicates stronger discounting (see Table 2). In the Probability Discounting for Gains and Probability Discounting for Losses tasks, gambles were played with five probabilities (2/3, 1/2, 1/3, 1/4, 1/5), and 30 trials with amounts of €5–30 were offered. Participants chose between a sure gain or loss and the probability of winning or losing a bigger amount of money (e.g., winning €5 for sure or winning €25 with a 20% probability). In the Probability Discounting for Gains task, risk-aversion is defined as the preference for the sure amount over the probabilistic one, reflected by higher k values (see Table 2). By contrast, the Probability Discounting for Losses task produces higher k values when individuals are more risk-seeking and prefer the probabilistic offer over the certain one (see Table 2). In the Mixed Gambles task, 40 gambles with a 50% chance of winning (€1–40) or losing (€5–20) were offered (e.g., rejecting to gamble or gambling for winning €15 or losing €8). Higher λ values indicate weighing losses relatively higher and tending to reject gambles (see Table 2). As people may show greater sensitivity to losses than to equivalent gains, participants received €10 before performing the Mixed Gambles task to promote gambling^38^.

**Table 2:** Value functions for modeling and parameter estimations of value-based decision-making tasks: V (subjective value of offer), A (amount of offer), k (discounting rate), D (length of delay [days]), p (probability of winning in Probability Discounting for Gains task or losing in Probability Discounting for Losses task), G (amount of gain), λ (loss aversion parameter), L (amount of loss). Adapted from Pooseh *et al*.^36^.

Value-based decision-making task	Equation
Delay Discounting	V = A/(1 + k D)
Probability Discounting for Gains/Losses	V = A/(1 + k [1 − p]/p)
Mixed Gambles	V = 1/2 (G − λ L)

The test battery uses an adaptive Bayesian algorithm and makes offers that are close to each individual’s estimated indifference point based on his or her previous choices^36^. The indifference point is reached when an individual judges both offers to be of equivalent subjective values. The estimated k/λ is used as the measure of impulsive choice for the respective task. The number of trials applied for each task guarantees achieving stable estimations^36^.

### Statistical analyses

We used SPSS Statistics Version 25 (IBM, Armonk, NY, USA) and assumed two-tailed significance at p < 0.05 for all analyses. We plotted histograms and normal quantile-quantile plots to check for normality. To meet the assumptions of parametric testing, we used natural log transformations of k and λ. We carried out repeated measures analysis of covariance (ANCOVA) for each task and applied partial η^2^ as a measure of effect size (small = 1%, medium = 6%, large = 14%)^39^. We used k/λ for both drug conditions as within-subject variables. Drug order was used as between-subjects factor because some participants received placebo and others L-DOPA first (crossover design). To test whether L-DOPA has baseline-dependent effects, BIS-15 total score was centered on the mean and considered as a covariate. Because of study observations that L-DOPA effects depend on body weight^19,20^, the above-named analyses were repeated using weight group (defined by median split of body weight at L-DOPA visit) as another between-subjects factor, and also were run separately for low- and high-weight participants. In exploratory analyses, we included sex as an additional between-subjects factor. We conducted a sensitivity power analysis using G*Power Version 3.1.9.2 (www.gpower.hhu.de) to calculate the critical population effect size to find main effects accepting a type II error probability of 20%^40^. Our whole sample (N = 87) was sufficiently powered to detect a small to medium main effect (d_z_ = 0.304). We used Pearson’s r to characterize the correlations between: 1) k/λ of the placebo and L-DOPA condition of each task in the test battery, 2) k/λ of different tasks, 3) k/λ and BIS-15 total score and 4) BIS-15 and sex. Data after placebo from one participant on the Mixed Gambles task were lost.

### Ethics

This study was approved by the institutional review board of the Technische Universität Dresden (EK 44022012) and the German Federal Office for Radiation Protection. The methods were performed in accordance with relevant guidelines and regulations. All participants gave written informed consent.

## Results

Repeated measures ANCOVA showed no significant differences between placebo and L-DOPA conditions for delay discounting (log k_DD_), risk-seeking for gains (log k_PDG_) and losses (log k_PDL_), or for loss aversion (log λ_MG_) (i.e., no main effect of medication on any of the four parameters of impulsive choice). There were no significant interactions between drug condition and drug order (i.e., no effect of repeating any of the tasks). Using body weight grouping also did not reveal main effects of L-DOPA on impulsive choice (see Table 3 and Supplementary Tables S1, S2). Sex as additional between-subjects factor was not associated with decision-making parameters.

**Table 3:** Repeated measures ANCOVA: K/λ for both drug conditions were used as within-subject variables.

		All subjects (N = 87 [DD, PDG, PDL], N = 86 [MG])			Low-weight subjects (N = 44 [DD, PDG, PDL], N = 43 [MG])		
		F	p	partial η²	F	p	partial η²
Delay discounting (log k_DD_)	Drug	0.501	0.481	0.006	0.061	0.806	0.001
	Drug × BIS-15	2.443	0.122	0.028	4.168	0.048*	0.092
	Drug order	0.475	0.493	0.006	0.655	0.423	0.016
	Drug × Drug order	0.916	0.341	0.011	1.522	0.224	0.036
	Intercept	398.982	0.000*	0.826	209.015	0.000*	0.836
Risk-seeking for gains (log k_PDG_)	Drug	0.395	0.532	0.005	0.008	0.927	0.000
	Drug × BIS-15	4.064	0.047*	0.046	4.469	0.041*	0.098
	Drug order	1.435	0.234	0.017	5.879	0.020*	0.125
	Drug × Drug order	0.635	0.428	0.008	0.852	0.361	0.020
	Intercept	3.576	0.062	0.041	6.516	0.015*	0.137
Risk-seeking for losses (log k_PDL_)	Drug	0.741	0.392	0.009	0.019	0.891	0.000
	Drug × BIS-15	2.626	0.109	0.030	0.033	0.858	0.001
	Drug order	0.015	0.903	0.000	0.069	0.795	0.002
	Drug × Drug order	0.384	0.537	0.005	0.512	0.478	0.012
	Intercept	2.493	0.118	0.029	0.220	0.642	0.005
Loss aversion (log λ_MG_)	Drug	0.264	0.609	0.003	0.035	0.852	0.001
	Drug × BIS-15	1.530	0.220	0.018	5.136	0.029*	0.114
	Drug order	2.612	0.110	0.031	6.585	0.014*	0.141
	Drug × Drug order	0.367	0.547	0.004	0.001	0.971	0.000
	Intercept	0.055	0.814	0.001	0.080	0.779	0.002

Drug order was used as between-subjects factor because some participants received placebo and others L-DOPA first (crossover design). BIS-15 total score was considered as covariate. *p < 0.05.

**Figure 1:**
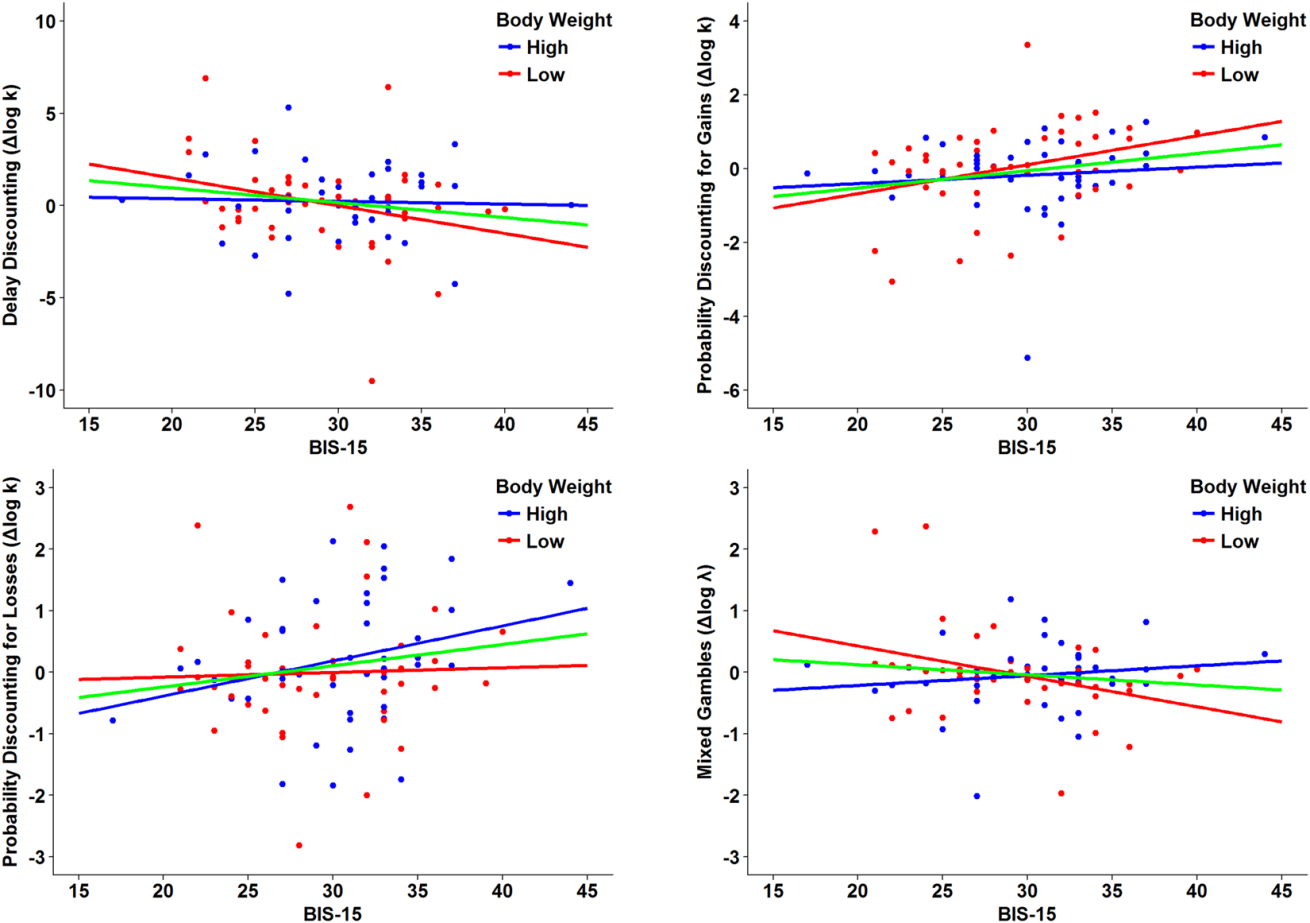
Relationship between change in value-based decision-making tasks by L-DOPA and BIS-15 total score: Δlog k/λ = log k/λ (L-DOPA) −log k/λ (Placebo). Each dot represents an individual subject, who was grouped according to body weight based on a median split (≤80.5 kg). The blue and red regression lines refer to the weight-grouped subsets, whereas the green line pertains to the whole sample.

In the whole sample (N = 87), the interaction between BIS-15 and drug condition significantly predicted risk-seeking for gains as measured by the Probability Discounting for Gains task (log k_PDG_, p = 0.047, partial η^2^ = 4.6%). Decision-making parameters of the other tasks were not affected (see Table 3). However, there was a significant three-way interaction between BIS-15, drug condition and weight group on loss aversion behavior (log λ_MG_, p = 0.022, partial η^2^ = 6.3%), and a trend-level three-way interaction on delay discounting (log k_DD_, p = 0.127, partial η^2^ = 2.8%) (Supplementary Table S2). When restricting the analyses to the 44 low-weight subjects (≤80.5 kg), the interaction between baseline impulsivity (BIS-15) and drug condition also significantly predicted delay discounting behavior (log k_DD_, p = 0.048, partial η^2^ = 9.2%) as well as loss aversion (log λ_MG_, p = 0.029, partial η^2^ = 11.4%), and had a significant effect on risk-seeking for gains (log k_PDG_, p = 0.041, partial η^2^ = 9.8%) (see Table 3). This result implies that changes in choice behavior on the Delay Discounting, Probability Discounting for Gains and Mixed Gambles tasks depended on baseline impulsivity: participants with lower BIS-15 scores discounted delays more strongly (increased k_DD_), and became more risk-seeking for gains (decreased k_PDG_) and more loss averse (increased λ_MG_) after having taken L-DOPA, whereas the opposite was detected for participants with higher BIS-15 scores (see Fig. 1). Sex did not explain this weight-dependent effect. Significant drug order effects observed in low-weight subjects with respect to risk-seeking for gains (log k_PDG_) and loss aversion (log λ_MG_) may be due to an increased likelihood that some participant characteristics were not evenly distributed in the subsample. However, there were no significant interactions between drug condition and drug order (see Table 3). Decision-making parameters were not affected in high-weight participants (Supplementary Table S1).

The k/λ parameters in the placebo and L-DOPA conditions (for raw values see Supplementary Table S3) were significantly correlated within the same tasks, indicating fair to good test-retest reliability (Supplementary Table S4). There were, however, only some weak correlations (largest r = 0.286) between performance on the different tasks: 1) log k_DD_ and log k_PDL_ correlated inversely within the L-DOPA condition, 2) log k_PDG_ of the placebo condition correlated positively with log λ_MG_ of both conditions and 3) log k_PDG_ of the L-DOPA condition correlated positively with log k_PDL_ of both conditions (Supplementary Table S4). There were no significant correlations between task performance and BIS-15 scores (Supplementary Table S4). BIS-15 was also not significantly correlated to sex (whole sample: r_pb_ = −0.015, p = 0.889; low-weight subset: r_pb_ = −0.096, p = 0.536).

## Discussion

Our goal was to clarify the role of dopamine in impulsive choice by investigating the effects of L-DOPA on four facets of value-based decision-making. In line with our prediction, L-DOPA had no main effect on impulsive choice, but had an effect on risk-seeking for gains, which was moderated by baseline impulsivity as assessed with the BIS-15. A post-hoc analysis in low-weight subjects suggests such a baseline-dependent effect also on delay discounting and loss aversion. After L-DOPA intake, more-impulsive individuals became less impulsive but low-impulsive individuals made more impulsive choices on the Delay Discounting and Probability Discounting for Gains tasks. The effect on loss aversion (Mixed Gambles task), however, seems difficult to reconcile with these results as impulsive participants became even less loss averse with L-DOPA, whereas low-impulsive individuals showed a further increase in loss aversion.

In light of previous studies showing paradoxical effects of drugs affecting dopamine signaling, our results suggest an inverted U-shaped relation between impulsivity and dopamine, which is already well established for working memory and helped to resolve the large variability of findings in that field^24,41^. An inverted U-shaped influence of dopamine has previously been suggested for delay discounting on the basis of tolcapone effects in 23 healthy adults^26^. Our results extend this finding, using a different drug in a much larger sample of healthy adults, and testing three dimensions of impulsive choice. Low-impulsive individuals (presumed optimal dopaminergic signaling) apparently were overdosed with L-DOPA, exhibiting increased delay discounting, risk-seeking for gains and loss aversion, whereas the opposite was detected for more-impulsive individuals (presumed suboptimal baseline dopaminergic signaling). Although this interpretation is speculative because our study design did not allow for a proper testing of an inverted U-shaped function, the effects in low-impulsive subjects are completely in line with predictions from a computational framework on dopaminergic function^8^. This framework postulates that tonic dopaminergic signaling codes an overall average outcome expectation against which all outcomes are compared. This “baseline” would linearly increase or decrease the opportunity costs and the perceived value of an outcome during decision-making and thereby determine which potential outcome is preferred. Our results indicate that the proposed linear influences are limited to individuals who have optimal dopaminergic transmission and get overdosed. Evidence from a genetic study of performance on the Balloon Analogue Risk Task supports our hypothesis of an inverted U-shaped association of dopaminergic function, with impaired performance at low and high extremes of dopaminergic function^25^. Striatal dopaminergic signaling was assessed using a composite score of functional polymorphisms across five genes encoding dopamine receptors (D2, D3, D4), the dopamine transporter (DAT1) and COMT. Further research might help to integrate these results and develop a more comprehensive computational model of dopaminergic function considering that animal studies suggest a family of functions (e.g., biphasic, sigmoidal, exponential) that might describe dopaminergic activity across different decision-making processes^42^.

We hypothesized that impulsive individuals would get less impulsive, whereas low-impulsive individuals would get more impulsive when administered L-DOPA. Interestingly, impulsive participants became less loss averse in mixed gambles, whereas low-impulsive individuals showed a further increase in loss aversion. A rather counterintuitive connection between high impulsivity and high loss aversion has already been demonstrated in patients with gambling disorder, who were more impulsive than healthy controls, as assessed with BIS-11 (motor and non-planning subscales) and more loss averse in mixed gambles^43^. As further analysis revealed that only patients in the later stages of treatment were more loss averse than controls, the difference may be related to treatment^43^. Since BIS-15 results were not significantly correlated to our Mixed Gambles task, it is at present unclear how to reconcile these findings.

We predicted that high-impulsive individuals would also become less risk-seeking for losses after L-DOPA administration, whereas those with lower impulsivity at baseline would then make more impulsive choices after receiving L-DOPA. Although this effect was not shown in the full sample or in the subset of low-weight individuals, this negative finding is in line with the result of a previous study^19^. Moreover, our results are consistent with a genetic study in which carriers of the 9-repeat allele of DAT1 (lower dopaminergic tone) were more risk-tolerant over gains than those with the 10-repeat allele (presumed higher dopaminergic tone)^44^. Notably baseline impulsivity was not considered in these studies.

Apart from investigating the impact of L-DOPA on each task, we explored how these tasks are related to one another. Similar to previous studies^4^, we observed only subtle correlations between performance on the tasks. Thus, we assume that we studied distinct facets of impulsive choice^4,32^. The lack of significant correlations between performance on value-based decision-making tasks and self-reports on the BIS-15 highlight once more the current understanding of impulsivity as a multidimensional trait^45,46^. As the decision-making parameters obtained were comparable to previous reports^10,17,38,43,46–48^, we are confident that our tasks worked as intended.

One limitation of this study is that we administered a fixed dose of L-DOPA to our participants. Including participants with a large range in body weight (49–128 kg) might have contributed to insufficient L-DOPA levels in high-weight participants, possibly reducing the effects of L-DOPA in our study. Notably, a previous study found that the efficacy of L-DOPA dose depends on body weight: the higher the L-DOPA dose per kg body weight, the higher was the increase in risk-seeking for gains with L-DOPA compared to placebo^19^. We, therefore, repeated our analyses using weight grouping and found that body weight mediated the baseline-dependent effect on loss aversion. Further analyses revealed that decision-making parameters were not affected in high-weight participants, but that low-weight subjects exhibited a baseline-dependent effect not only on risk-seeking for gains but also on loss aversion and delay discounting. These results tentatively suggest that modifying L-DOPA doses according to body weight might have revealed baseline-dependent effects on delay discounting and loss aversion in the full sample. Another possible limitation was that our sample had BIS-15 scores of 17 to 44 (low-weight group: 21–40) from a possible range of 15 to 60, with very impulsive individuals not represented. This could have further limited the power to detect baseline-impulsivity dependence and associations between self-report of impulsivity and performance on value-based decision-making tasks.

In conclusion, our results suggest that the relationship between dopamine signaling and impulsive decision-making may be nonlinear but follows an inverted U-shaped function, and that the response to L-DOPA dose depends on body weight. Yet our findings would not have survived correction for multiple comparisons, and must therefore be interpreted as preliminary. Future PET studies that measure baseline dopamine availability and compare effects of dopaminergic drugs and doses could provide stronger evidence for nonlinear effects. The consideration of pharmacological effects as related to baseline impulsivity could improve our understanding of the underlying mechanisms of impulsivity. As impulsivity is an important feature of many psychiatric disorders, studying how dopamine signaling is related to impulsivity in patients can deepen our knowledge of these diseases.

## Supplementary information

Supplementary Information

## Data Availability

All data generated and analyzed during this study are available from the corresponding author on reasonable request.
