# Supplementary material for: Baseline impulsivity may moderate L-DOPA effects on value-based decision-making
Source: Sci Rep. 2019 Apr 4;9:5652. doi: 10.1038/s41598-019-42124-x (PMC6449394; doi:10.1038/s41598-019-42124-x)
Supplement: Supplementary file 1 — Supplementary Information [file 41598_2019_42124_MOESM1_ESM.docx]

# Supplementary Information

Baseline impulsivity may moderate L-DOPA effects on value-based decision-making

Johannes Petzold, MD; Annika Kienast; Ying Lee; Shakoor Pooseh, PhD; Edythe D London, PhD; Thomas Goschke, PhD; Michael N Smolka, MD

**Table S1** Repeated measures ANCOVA: K/λ for both drug conditions were used as within-subject variables. Drug order was used as between-subjects factor because some participants received placebo and others L-DOPA first (crossover design). BIS-15 total score was considered as covariate. N = 43. * (p < 0.05).

|  |  | **High-weight subjects** | | |
| --- | --- | --- | --- | --- |
|  |  | F | p | partial η² |
| **Delay discounting**  (log k_DD_) | Drug | 0.564 | 0.457 | 0.014 |
|  | Drug × BIS-15 | 0.025 | 0.875 | 0.001 |
|  | Drug order | 0.075 | 0.785 | 0.002 |
|  | Drug × Drug order | 0.124 | 0.726 | 0.003 |
|  | Intercept | 180.196 | 0.000* | 0.818 |
| **Risk-seeking for**  **gains** (log k_PDG_) | Drug | 1.228 | 0.274 | 0.030 |
|  | Drug × BIS-15 | 0.407 | 0.527 | 0.010 |
|  | Drug order | 1.605 | 0.213 | 0.039 |
|  | Drug × Drug order | 0.033 | 0.856 | 0.001 |
|  | Intercept | 0.095 | 0.759 | 0.002 |
| **Risk-seeking for**  **losses** (log k_PDL_) | Drug | 1.798 | 0.187 | 0.043 |
|  | Drug × BIS-15 | 3.540 | 0.067 | 0.081 |
|  | Drug order | 0.747 | 0.392 | 0.018 |
|  | Drug × Drug order | 0.206 | 0.653 | 0.005 |
|  | Intercept | 3.325 | 0.076 | 0.077 |
| **Loss aversion**  (log λ_MG_) | Drug | 0.378 | 0.542 | 0.009 |
|  | Drug × BIS-15 | 0.695 | 0.410 | 0.017 |
|  | Drug order | 1.347 | 0.253 | 0.033 |
|  | Drug × Drug order | 0.192 | 0.664 | 0.005 |
|  | Intercept | 0.124 | 0.727 | 0.003 |

**Table S2** Repeated measures ANCOVA: K/λ for both drug conditions were used as within-subject variables. As between-subjects factors we used body weight group (defined by median split) to account for possible weight-dependent L-DOPA effects and drug order (crossover design). BIS-15 total score was considered as covariate. N = 87 (missing data after placebo from one participant on MG). * (p < 0.05).

|  |  | **All subjects** | | |
| --- | --- | --- | --- | --- |
|  |  | F | p | partial η² |
| **Delay discounting**  (log k_DD_) | Drug | 0.244 | 0.623 | 0.003 |
|  | Drug × BIS-15 | 2.540 | 0.115 | 0.030 |
|  | Drug × BIS-15 × Weight group | 2.374 | 0.127 | 0.028 |
|  | Weight group | 0.067 | 0.796 | 0.001 |
|  | Drug × Weight group | 0.209 | 0.649 | 0.003 |
|  | Drug order | 0.577 | 0.450 | 0.007 |
|  | Drug × Drug order | 1.437 | 0.234 | 0.017 |
|  | Intercept | 387.977 | 0.000* | 0.826 |
| **Risk-seeking for**  **gains** (log k_PDG_) | Drug | 0.236 | 0.629 | 0.003 |
|  | Drug × BIS-15 | 4.550 | 0.036* | 0.053 |
|  | Drug × BIS-15 × Weight group | 1.152 | 0.286 | 0.014 |
|  | Weight group | 4.423 | 0.039* | 0.051 |
|  | Drug × Weight group | 1.241 | 0.269 | 0.015 |
|  | Drug order | 1.263 | 0.264 | 0.015 |
|  | Drug × Drug order | 0.333 | 0.566 | 0.004 |
|  | Intercept | 3.447 | 0.067 | 0.040 |
| **Risk-seeking for**  **losses** (log k_PDL_) | Drug | 0.476 | 0.492 | 0.006 |
|  | Drug × BIS-15 | 2.303 | 0.133 | 0.027 |
|  | Drug × BIS-15 × Weight group | 1.521 | 0.221 | 0.018 |
|  | Weight group | 0.891 | 0.348 | 0.011 |
|  | Drug × Weight group | 0.694 | 0.407 | 0.008 |
|  | Drug order | 0.183 | 0.670 | 0.002 |
|  | Drug × Drug order | 0.697 | 0.406 | 0.008 |
|  | Intercept | 1.812 | 0.182 | 0.022 |
| **Loss aversion**  (log λ_MG_) | Drug | 0.670 | 0.415 | 0.008 |
|  | Drug × BIS-15 | 1.547 | 0.217 | 0.019 |
|  | Drug × BIS-15 × Weight group | 5.434 | 0.022* | 0.063 |
|  | Weight group | 0.216 | 0.644 | 0.003 |
|  | Drug × Weight group | 0.006 | 0.938 | 0.000 |
|  | Drug order | 2.193 | 0.142 | 0.026 |
|  | Drug × Drug order | 0.089 | 0.766 | 0.001 |
|  | Intercept | 0.092 | 0.763 | 0.001 |

**Table S3** k/λ of placebo and L-DOPA condition of each task in the test battery (delay discounting [k_DD_], risk-seeking for gains [k_PDG_], risk-seeking for losses [k_PDL_], loss aversion [λ_MG_]). N = 87 (missing data after placebo from one participant on MG).

|  |  | k_DD_ | k_PDG_ | k_PDL_ | λ_MG_ |
| --- | --- | --- | --- | --- | --- |
| Mean ± SD | Placebo | 0.20 ± 1.14 | 2.38 ± 5.00 | 1.41 ± 2.28 | 1.33 ± 1.00 |
|  | L-DOPA | 0.06 ± 0.17 | 1.58 ± 1.56 | 1.70 ± 2.69 | 1.23 ± 0.80 |

**Table S4** Correlations between: 1) k/λ of placebo and L-DOPA condition of each task in the test battery (delay discounting [log k_DD_], risk-seeking for gains [log k_PDG_], risk-seeking for losses [log k_PDL_], loss aversion [log λ_MG_]), 2) k/λ of different tasks, 3) k/λ and BIS-15 total score. Pearson’s r was used as linear correlation coefficient. P values are in parentheses. N = 87 (missing data after placebo from one participant on MG). * (p < 0.05).

|  |  | Log k_DD_ | | Log k_PDG_ | | Log k_PDL_ | | Log λ_MG_ | |
| --- | --- | --- | --- | --- | --- | --- | --- | --- | --- |
|  |  | Placebo | L-DOPA | Placebo | L-DOPA | Placebo | L-DOPA | Placebo | L-DOPA |
| BIS-15 |  | 0.142 (0.189) | 0.001 (0.996) | -0.195 (0.071) | 0.042 (0.702) | -0.133 (0.219) | 0.020 (0.854) | -0.012 (0.913) | -0.117 (0.282) |
| Log k_DD_ | Placebo |  | *0.646 (0.000) | -0.027 (0.803) | -0.119 (0.274) | 0.071 (0.516) | -0.119 (0.274) | -0.136 (0.213) | -0.064 (0.558) |
|  | L-DOPA |  |  | 0.049 (0.653) | -0.125 (0.247) | -0.050 (0.644) | *-0.220 (0.041) | -0.198 (0.068) | -0.055 (0.612) |
| Log k_PDG_ | Placebo |  |  |  | *0.321 (0.002) | -0.016 (0.882) | -0.067 (0.535) | *0.235 (0.030) | *0.286 (0.007) |
|  | L-DOPA |  |  |  |  | *0.216 (0.045) | *0.230 (0.032) | 0.207 (0.056) | 0.162 (0.134) |
| Log k_PDL_ | Placebo |  |  |  |  |  | *0.611 (0.000) | 0.018 (0.868) | 0.019 (0.862) |
|  | L-DOPA |  |  |  |  |  |  | -0.029 (0.793) | -0.167 (0.123) |
| Log λ_MG_ | Placebo |  |  |  |  |  |  |  | *0.645 (0.000) |
|  | L-DOPA |  |  |  |  |  |  |  |  |
